# An insight into the transmission role of insect vectors based on the examination of gene characteristics of African swine fever virus originated from non-blood sucking flies in pig farm environments

**DOI:** 10.1186/s12917-020-02420-5

**Published:** 2020-07-02

**Authors:** Jinling Liu, Gen Lu, Yuesong Cui, Shu Wei, Tongqing An, Guoshun Shen, Zeliang Chen

**Affiliations:** 1 Key Laboratory of Livestock Infectious Diseases in Northeast China, Ministry of Education, College of Animal Science & Veterinary Medicine, Shenyang Agricultural University, No.120, Dongling Road, Shenhe District, Shenyang, 110866 PR China; 2 The Preventive Center of Animal Disease of Liaoning Province, No.95, Renhe Road, Shenbei District, Shenyang, 110164 PR China; 3 Harbin Veterinary Research Institute of Chinese Academy of Agricultural Sciences, No. 678, Haping road, Xiangfang district, Harbin, 150069 PR China; 4 Beijing Advanced Innovation Center for Soft Matter Science and Engineering, Beijing University of Chemical Technology, Beijing, 100029 PR China; 5 Brucellosis Prevention and Treatment Engineering Technology Research Center of Inner Mongolia Autonomous region, Inner Mongolia University for Nationalities, Tongliao, 028000 PR China

**Keywords:** ASFV, Non-blood sucking fly, Differential gene, Variant, Transmission

## Abstract

**Background:**

Insect vector transmitted pathogens from contaminated environments are a key potential risk for public health. Meanwhile, transmission by non-blood sucking flies needs to be considered. Sequencing and phylogenetic tree analyses were used to study African swine fever virus (ASFV) genes derived from flies collected from pig farms that were infected with ASFV. The major differential genes were analyzed the encoded proteins, particularly their conformation, physico-chemical features, and interactions identified by immunophenotyping.

**Results:**

Results showed that the ASFV *p72* and *D117L* genes from these non-blood sucking flies identified by morphology have high sequence similarity from ASFV genotype II strains, however, A179L is found in an independent cluster, with five amino acid substitutions; four of which are in a continuous sequence. Moreover, the binding of a BH3 peptide into a surface groove formed by α-helices of ASFV A179L from the non-blood sucking flies is consistent with that of representative ASFV genotype II strains, Georgia/2007.They only differ in the direction of spatial interaction of six conserved amino residues. Many hydrophilic amino residues are located at the canonical ligand-binding groove of A179L from flies, with hydrophobic amino residues located at the corresponding positions in A179L of the Georgia/2007.Furthermore, analysis of protein interactions by immunophenotyping revealed that both A179Ls have similar roles in regulating autophagy and apoptosis.

**Conclusions:**

In conclusion, the main genes that differ between ASFV from flies and Georgia/2007 were similar in structure and protein interaction, while exhibiting differences in physico-chemical features and amino acid variations. Understanding the mechanical transmission characteristics of non-blood sucking flies is important.

## Background

African swine fever (ASF) is a notifiable, highly contagious, and fatal viral hemorrhagic fever that affects all species of the Suidae family. Currently, no safe vaccines or treatments are available for prevention and control [1]. ASF has a serious socioeconomic impact on the international trade of pigs and pig products [2–4]. On August 3, 2018, the first ASF case in China was reported in in the city of Shenyang of the Liaoning province. As of September 30, 2019, ASF outbreaks were reported in almost all geographical regions of China, affecting 31 provinces, and seriously impacting pig production and pork consumption. However, between August 2018 and September 2019, ASF outbreaks in China gradually decreased, indicating that the improvements in biosecurity measures on farms and more stringent surveillance by veterinary authorities have been effective (http://www.moa.gov.cn/xw/zwdt/201902/t201902 02_6171163.htm).

Concurrently, we noticed that most ASF outbreaks were detected during the warmer seasons [5]. Average mortality and morbidity rates were considerably higher between April and September in China (http://www. moa. gov.cn/xw/zwdt/201902/ t201902026171163.htm), implying the importance of seasonality in ASF transmission. Interestingly, the increasing numbers of ASF outbreaks observed in domestic herds during the summer period also coincided with increased insect prevalence in China, suggesting a potential role of insects as vectors for transmitting ASFV. It is well known from previous studies that ASFV transmission can also occur via animal contact, contaminated feed or fomites, as well as through tick vectors [6]. In particular, one recently study demonstrated that blood-sucking flies, such as stable flies (*Stomoxys calcitrans*), have been shown to transmit ASFV mechanically after ingesting viremia blood and subsequently feeding on pigs [7]. Currently, no studies have shown evidence of ASFV transmission by non-blood sucking flies [8]. To advance our understanding of such modes of transmission, here, we aimed to further elucidate the transmission characteristics of non-blood sucking flies in ASFV infection and to contribute toward an assessment of the potential public health risk posed by such flies.

## Results

### Sequence alignment and homology analysis

**Fig. 1:**
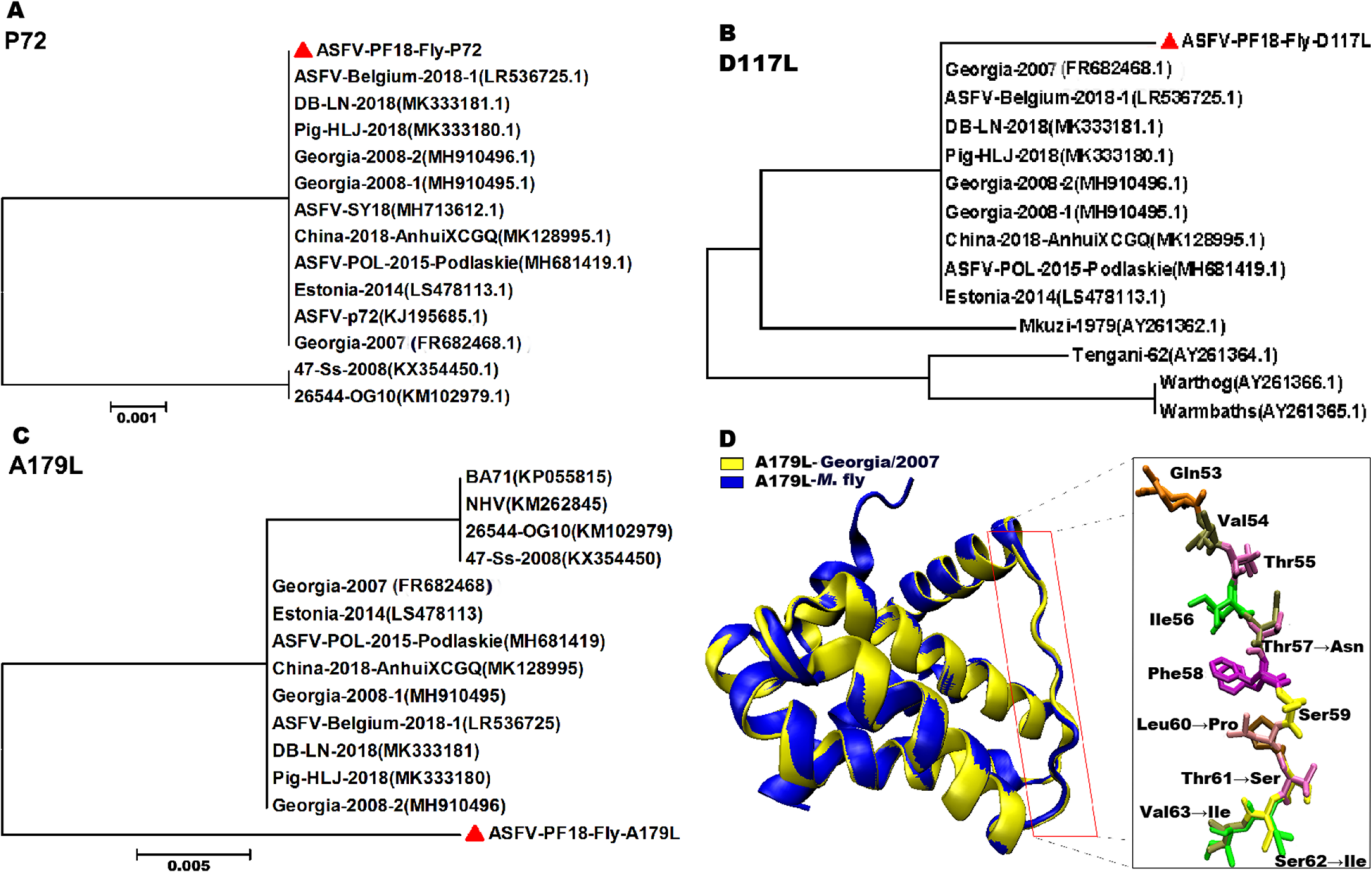
Phylogenetic analysis and amino acid sequence differentiation of ASFV genes from *M. domestica* flies and other ASFV strains. **a-c** Phylogenetic relationship of ASFV *P72, D117L, and A179L* genes originated from *M. domestica* flies and other ASFV strains. We retrieved 13 publicly available gene sequences of ASFV strains from NCBI. The GenBank accession numbers of these strains are included in the analyses and are indicated in parentheses. Sequences were aligned using MEGA 6.0, with bootstrapping of 1000 replicates. The gene sequences used in this study are marked with ▲. **d** The amino acid sequence of ASFV A179L originating from *M. domestica* flies differs from the representative genotype II strain, Georgia/2007, by five amino acid substitutions, observed by superimposing the structures of ASFV A179L from *M. domestica* flies and Georgia/2007, shown in yellow and blue, respectively. The circle shows the region where the different amino acids are located, and amino acid substitution sites are presented in different colors

Collecting flies (*n* = 35) were identified morphologically using identification keys such as color and stripe of fly body, chest and abdomen, wings and tentacles, etc., and those identified flies included *M. domestica* (*n* = 32) and *Drosophila spp.* (*n =* 3). Tested results of ASFV-DNA were positive for *M. domestica* flies and negative for *Drosophila* flies. Subsequently, the positive DNA products of PCR were sequenced. As shown in Fig. 1.a-b, the ASFV *p72 and D117L* genes from *M. domestica* flies clustered with other genotype II isolates, such as Georgia 2007/1, Georgia 2008–1/2, Belgium 2008/1, ASFV-Pol-2015-Podlaskie, Estonia-2014, and China 2018 isolates from Heilongjiang, Shenyang, and Anhui. ASFV genotype identification often depends on partial *p72* gene characterization. Thus, the ASFVs from *M. domestica* flies (ASFV-PF18-Fly) in this study belong to the genotype II (Fig. 1a). However, the *A179L* gene of ASFV-PF18-Fly was present in an independent cluster compared to the other 13 isolates analyzed in this study (Fig. 1c). Protein sequences of ASFV A179L from *M. domestica* flies and a representative strain of ASFV genotype II, Georgia/2007, were compared. As expected, the amino acid sequence encoded by A179L gene of ASFV from *M. domestica* flies differs from that of Georgia/2007 by five amino acid substitutions, observed by superimposing their corresponding protein structures. These substitutions include Thr for Asn at position 57, Leu for Pro at position 60, Thr for Ser at position 61, Ser for Ile at position 62, and Val for Ile at position 63. Positions 60–63 contain a variation of four consecutive amino acids (Fig. 1d).

### Comparing the structural configuration of ASFV A179L proteins

**Fig. 2:**
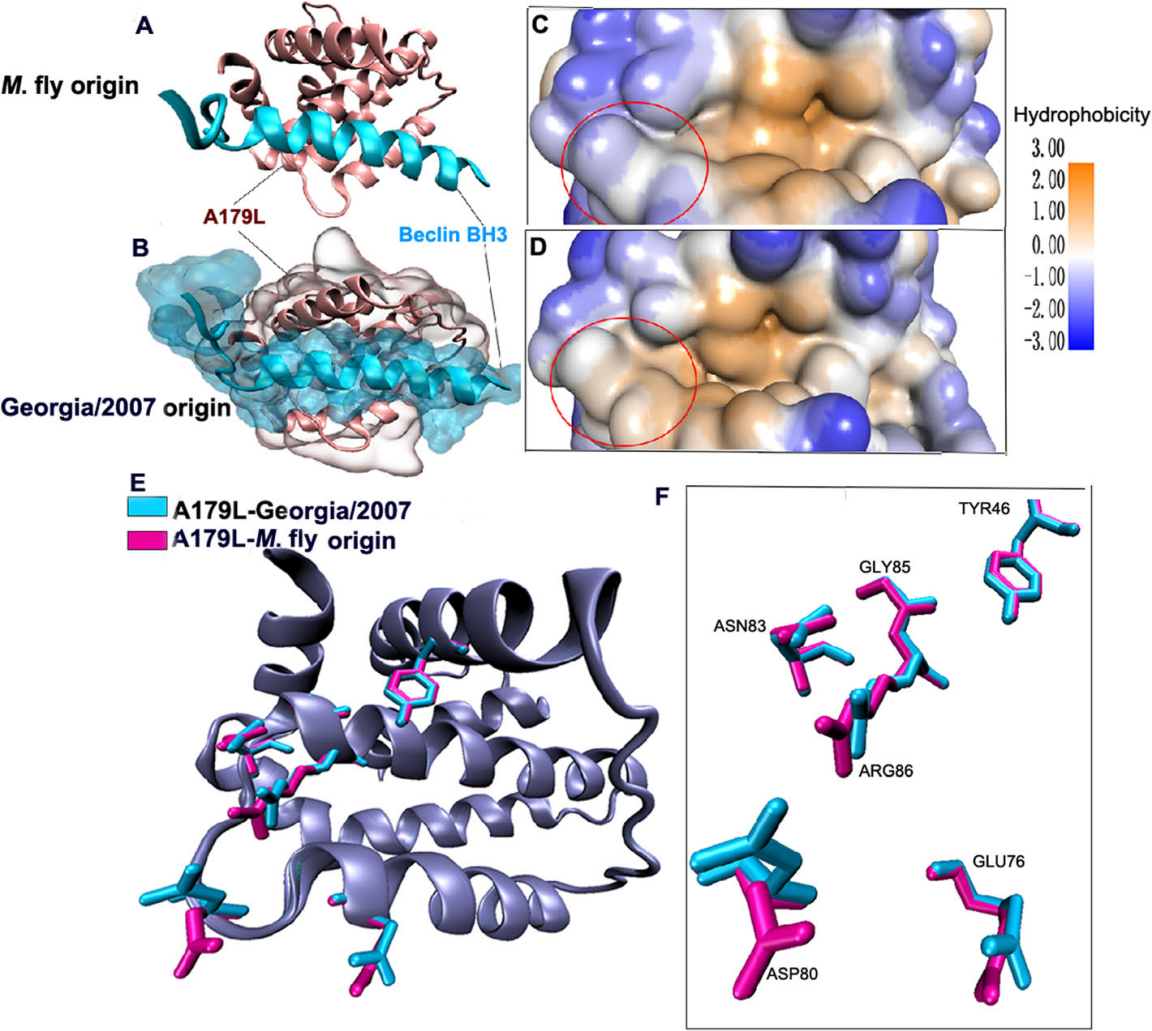
Detailed view of the A179L:BH3 peptide interfaces. **a** Beclin-BH3 peptide (light blue) binds to a groove formed by α-helices of ASFV A179L (brown) from *M. domestica* flies. **b** ASFV A179L from Georgia/2007 is shown in grey on the molecular surface, with the floor of the binding groove shown in brown. Beclin-BH3 is shown in light blue. **c-d** Hydrophobicity of ASFV A179L-BH3 protein structure. Different colors represent different degrees of hydrophobicity, ranging from + 3 to− 3. **c** Hydrophobic residues in yellow are distributed in the binding groove of ASFV A179L-BH3 from *M. domestica* flies. **d** Hydrophilic residues in blue are distributed in the binding groove of A179L-BH3from the Georgia/2007 strain. **e–f** The six conserved hydrophobic residues of Beclin-BH3 (Tyr46, Glu64, Asn83, Gly85, Arg80, and Asp86) are involved in binding grooves, but with different directions of interaction. Different colors represent amino acids from different species. Amino acids in ASFV A179L from Georgia/2007 and *M. domestica* flies are shown in light blue or light purple, respectively

The ASFV A179L configuration from *M. domestica* flies is similar to that of Georgia/2007, Both A179L proteins contain a BH3-binding motif in the surface groove formed by α-helices, where the BH3 peptide interacts with six conserved amino residues of A179L (Fig. 2a-b). While the key structural hallmarks of A179L-BH3 interaction are conserved in both A179L proteins, the six amino residues (Tyr46, Glu64, Asn83, Gly85, Asp80, and Arg86) that are responsible for stabilizing the complexes, which differ in the direction of their interactions (Fig. 2e-f). Beyond that, many hydrophilic amino residues are located at the central hydrophobic cleft of the ASFV A179L from *M. domestica* flies, while hydrophobic amino residues are distributed in the correspond location of A179L in the Georgia/2007 strain (Fig. 2c-d). Generally, protein receptors can recognize aromatic residues in deep hydrophobic cavities or through large clusters that form hydrophobic surfaces. Hydrophobic interactions play an important role in the initiation and propagation of protein folding [9].

### Immunophenotypes analysis of interaction partner with A179L homology

**Fig. 3:**
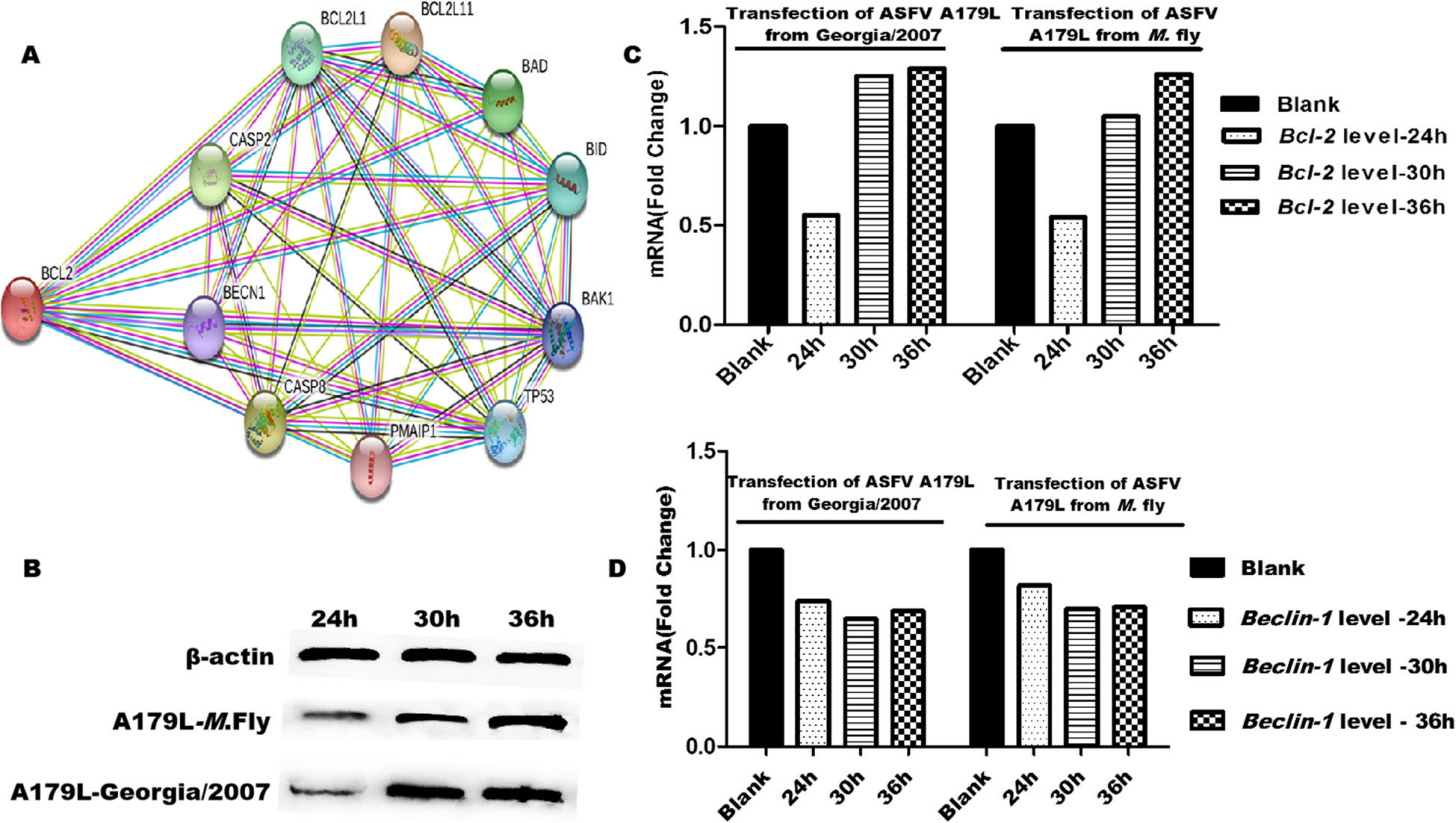
Protein interaction and gene expression analysis. An interactome map was constructed for the Bcl-2 protein, a homolog of A179L. Potential interaction partners of Bcl-2 with a high confidence score (C0.700) were predicted by STRING tool and verified by transfection and fluorescence qPCR. **a** Predicted map of interaction partners between Bcl-2 protein and A179L homolog from swine/pig. **b** Western blot analysis (cropping gels) of protein expression at different times after ASFV PEGX-A179L transfection into HEK-293 T cells. **c***Bcl-2* mRNA transcription levels at different times after ASFV PEGX- A179L from *M. domestica* flies and Georgia/2007 being transfected into HEK-293 T cells, respectively**. d***Beclin1* mRNA transcription levels at different times after ASFV PEGX-A179L from *M. domestica* flies and Georgia/2007 being transfected into HEK-293 T cells, respectively

An interactome map was constructed for Bcl-2, the A179L protein homolog. Potential interaction partners of Bcl-2 with a high confidence score (C0.700) were predicted using the STRING analysis tool. Analysis of protein-protein interactions showed that Bcl-2 can directly interact with Beclin1 in the pig internal environment, whereas more regulatory proteins such as BAD and BID also participate in the regulation (Fig. 3a). Thus, the transcription levels of *Bcl-2* and *Beclin1* were detected using fluorescent qPCR after transfecting ASFV *PEGX-A179L* from *M. domestica* flies and Georgia/2007 into HEK-293 T cells, respectively (Fig. 3b-d). The results indicated that over-expressions of A179L correlate with transcription levels of autophagy and apoptosis. Here, A179L from *M. domestica* flies and Georgia/2007 inhibited *Beclin-1* transcription, and up-regulated *Bcl-2* transcription at 30 h and 36 h, while down regulated it at 24 h (Fig. 3c-d).

## Discussion

As stated previously,various blood-sucking insects, such as mosquitoes are known to transmit viral and bacterial infections, as well as stable flies (*Stomoxys calcitrans*) and horse flies (*Tabanidae*) can transmit virus via being eaten by pigs during feeding or bites. Therefore, blood-sucking fling insects may play a role in pathogen transmission within farms. However, the flies caught in our study were *M. domestica* and *Drosophila spp.*, belong to non-blood sucking species. At present, no information exists about transmission of ASFV by these fly species. Additionally, prior studies that have noted the correlation between vector abundance and disease occurrence [10]. Hence, one possible route of ASFV introduction into farms might be via the flies that have traveled or been introduced from infected farms. This mode of transmission in domestic pigs could explain the increasing number of outbreaks observed during the summer months in domestic pigs in China in 2018–2019. It is unlikely that this route is the principal mode of viral transmission within pig herds, but it is possible mechanism for initial entry of the virus into a pig population on a farm.

In this study, another important finding was that we did not detect ASFV genes from *Drosophila* flies (*n* = 3), this may be due to the small sample size. Meanwhile, ASFV genes in *M. domestica* flies were detected using nest PCR not conventional PCR, indicating that the DNA content of ASFV from *M. domestica* flies is relatively low. Therefore, preliminary speculation was that flies were not biological host of ASFV, since ASFV replication within them is not continuous [11–13],when ASF viruses were ingested by *M. domestica* flies along with decaying food, or were mechanical transmitted by *M. domestica* flies after released into the environment in pig farm caused by infected pigs. However, this does not mean that ASFV is inactivated from all flies in proximity to pig farms in China. Although it is to be determined whether the ASFV from the flies represents an active infection or a dietary/environmental origin, it is worth considering that *M. domestica* flies may potentially contaminate pig-feeding sources or accidentally be ingested by pigs via carrying the virus on/in their bodies.

Moreover, discovery of a novel genotype XXIV that was reported for the first time in soft ticks in Mozambique, highlights the diversity of ASFV variants found in the sylvatic cycle [14]. In addition, it has previously been shown that the virus circulating in Sardinia has undergone genetic variation in two genome regions, B602L and EP402R. This variant has rapidly replaced the earliest viruses, perhaps because of some selective advantage, although clinical data suggest that Sardinian ASFV had no changes in virulence [15]. These studies highlight the epidemiological importance of biting vector transmission and the sylvatic cycle in harboring and disseminating new and existing virus strains, and broaden our understanding of the potential ecological and biological drivers affecting ASFV genetic variability present in the environment.

Generally, conserved open reading frames encode structural proteins; transcription factors, such as mammary gland specific nuclear factor (MGF) implicated in interferon type 1 (IFN I) immune response; or proteins involved in nucleotide metabolism, DNA repair, and viral replication, such as A240L [15, 16]. An inner envelope component, the protein p17 (encoded by D117L), is required for the assembly of the capsid layer on the membrane [17, 18]. A179L, the viral Bcl-2 homolog of ASFV, interacts with pro-apoptotic Bcl-2 family proteins to inhibit apoptosis, and affects autophagy by interacting with Beclin1 through its BH3 homology domain [19]. This domain displays major variability with five amino acid substitutions, four of which are in a continuous sequence. The degree of variation significantly differs from the other ASFV genes examined from *M. domestica* flies. Also, the other differences mainly display in the hydrophilicity of the BH3 binding groove and key conserved structural hallmarks of A179L-BH3 interaction features. The biological significance of these variations is not clear because we have not isolated the virus from these flies due to experiment condition limitation. Therefore, it is not clear whether or not the changes to protein structures interfere with their functions, the antigenic properties of A179L or affecting viral virulence, or more characteristics of non-blood sucking flies in transmission of ASFV.

As previously reported, ASFV A179L also inhibited autophagy by binding Beclin1 in the arthropod hosts [19, 20]. Protein interaction analysis using STING tool revealed that Bcl-2, the A179L homolog, can directly interact with Beclin1 in pig internal environment, whereas more regulatory proteins such as BAD and BID also participate in the regulation of the pig immune system. Concurrently, the results of A179L transfection and fluorescent qPCR verification showed that that over-expression of ASFV A179L from *M. domestica* flies and Georgia/2007 correlate with transcription levels of autophagy and apoptosis. qPCR results showed that *Bcl-2* transcription was higher after 36 h of transfection of A179L obtained from both flies and pigs, compared with those of *Bcl-2* at 24 h and 30 h, meanwhile, *Beclin-1* transcription of autophagy gene keeping decreased all the time. Numerous large DNA viruses to subvert programmed cell death-based host defense systems typically use structural and functional homologs of Bcl-2. The A179 L protein is expressed in cells in both early and late stages post-infection with ASFV, but is not packaged into virus particles [18, 21]. This suggests that A179 L may be involved in inhibiting apoptosis throughout infection, but not during the earliest stages when viral cores enter the cytoplasm. Furthermore, apoptotic regulation by ASFV A179L from pigs and *M. domestica* flies need to be studied over time to understand the differences in apoptotic pathways and mechanisms.

We observed an independent cluster in our analysis as a result of variations in the ASFV *A179L* gene of *M. domestica* flies. Interestingly, many ASFV genes from *M. domestica* flies collected in two pig farms have genetic variations similar to the A179L, forming independent branches. Most of them encoded proteins involved in immunosuppression (Unpublished data, personal communication with professor Zeliangchen research team). The biological characteristics of the variations is not clear because live virus isolation is not attempted. Fly sample size and pig farm number with ASFV infection are limited in this study, but the authors propose that DNA variation of non ASFV p72 gene from *M. domestica* flies gives us a new insight on the potential risks to public health caused by gene recombination of ASFV in the environment, together with genetic variation and transmission of ASFV by non-blood sucking flies, which need to be carefully considered. In the future, further studies including increasing the research of other pathogens harbored by flying insects in farms of negative for ASF, samples size for *M. domestica* and *Drosophila* flies as well as other non-blood sucking fly species, especially considering that functional identification of other ASFV proteins derived from flying insects instead of primarily focused on single detection analysis of ASFV *p72* gene from environmental samples. Furthermore, our study highlights the need for investigating the potential risk of ASFV harbored by non-blood sucking flies involved in spreading into the local domestic cycle and international value chains of pork product. These factors are important indicators of pathogen transmission by non-blood sucking flying insects.

## Conclusion

In summary, the major differential genes of ASFV from *M. domestica* flies in infected pig farm environments and representative strains of ASFV genotype II, Georgia/2007, are similar in structure and immunophenotype of interaction partners, but different in physico-chemical features and amino acid variations. This data shared first a summary of the major differential gene variation characteristics of ASFV harbored by non-blood sucking flies. It is important to be aware of potential risks to public health from genetic recombination and genetic variability of ASFV in non-blood sucking flies, along with transmission characteristics of flying insect vectors in spreading pathogens. In particular, on affected pig farms, increased disinfection of breeding environments to prevent further ASF outbreaks warranted. Our data give a new insight into the characteristics of mechanical transmission pathogens by non-blood sucking flies. This study provides preliminary information that will be important for controlling ASFV transmission risks by non-blood sucking flying insects and prevention programs.

## Methods

### Nucleotide identification and phylogenetic tree analysis

Flies were collected using flytraps in the vicinity of two pig farms that were confirmed to be infected with ASFV. Flies were killed using chloroform for species identification. After separation the body parts of eight-ten at a time, nucleic acids were extracted from the fly samples using a DNA/RNA Virus Kit (Transgene Biotech, China) according to the manufacturer’s instructions, and subjected to conventional and nest PCR amplification. The *A179L, D117L*, and *B646L* (encoding the p72 capsid protein) gene fragments of ASFV were amplified using six pairs of primers and identified by nucleotide sequencing. Subsequently, the retrieved nucleotide sequences were aligned using Clustal W, and the phylogenetic trees were drawn using MEGA 7.0, with the maximum likelihood method for 1000 bootstrap values. Additionally, the differences in amino acid sequences of the major ASFV differential genes were observed in conjunction with the corresponding superimposed structures of ASFV proteins.

### Construction of the three-dimensional model and comparison of physico-chemical features for major differential genes

The main differential gene identified from sequencing and evolutionary tree analysis was A179L, which we studied further. As the three-dimensional structure of ASFV A179L protein from flies has not yet been elucidated, comparative and predicted modeling was used. To achieve this, the amino acid sequences of A179L from flies and the Georgia/2007 strain (GenBank: FR682468) were aligned using Visual Molecular Dynamics 1.9.3 and submitted to the Swiss-Model server to construct the A179L model using the alignment mode. During this process, the crystallographic structure mode of the BH3 binding motif in ASFV A179L (5UA4) obtained from the Protein Data Bank (PDB) (www.rcsb.org) was used as a template. In addition, the degree of hydrophobicity, hydrophilicity, and properties of functional amino acids were evaluated using Discovery Studio 3.5.

### Analysis of protein interaction and phenotypic verification

A179L is the viral Bcl2 homolog (vBcl2). Similar to Bcl-2, A179L protects cells from apoptosis, even when expressed in heterologous systems such as vaccinia or baculovirus [22–24]. In order to further understand the characteristics of ASFV A179L from the flies and Georgia 2007/1 involved in apoptosis regulation, the proteins interacting with Bcl-2 were analyzed using STRING (Search Tool for the Retrieval of Interacting Proteins, http://string-db.org/) by referring to the related signal information. This followed by immunophenotyping verification of protein interactions using fluorescence quantitative PCR (qPCR; Thermo Fisher Scientific, USA) after being transfected the recombinant gene of ASFV *PEGX-A179L* from the flies and Georgia 2007/1(gene synthesis from Shanghai Bioengineering Co., Ltd., China) into HEK-293 T cells with Lipofectamine 2000 (Invitrogen), respectively.

## Supplementary information

**Additional file 1.**

## Data Availability

All data generated and/or analyzed during this study are included in this manuscript. The raw data are available from the corresponding author on reasonable request.

## Abbreviations

ASF: African swine fever
ASFV: African swine fever virus
DNA: Deoxyribonucleic Acid
PCR: Polymerase Chain Reaction
qPCR: quantitative PCR
STRING: Search Tool for the Retrieval of Interacting Proteins
HEK-293 T: Human Embryonic Kidney 293 T
PDB: Protein Data Bank
MEGA 7.0: Molecular Evolutionary Genetics Analysis 7.0
