# Supplementary material for: An insight into the transmission role of insect vectors based on the examination of gene characteristics of African swine fever virus originated from non-blood sucking flies in pig farm environments
Source: BMC Vet Res. 2020 Jul 2;16:227. doi: 10.1186/s12917-020-02420-5 (PMC7331130; doi:10.1186/s12917-020-02420-5)
Supplement: Supplementary file 1 — Additional file 1. [file 12917_2020_2420_MOESM1_ESM.pptx]

## Slide 1
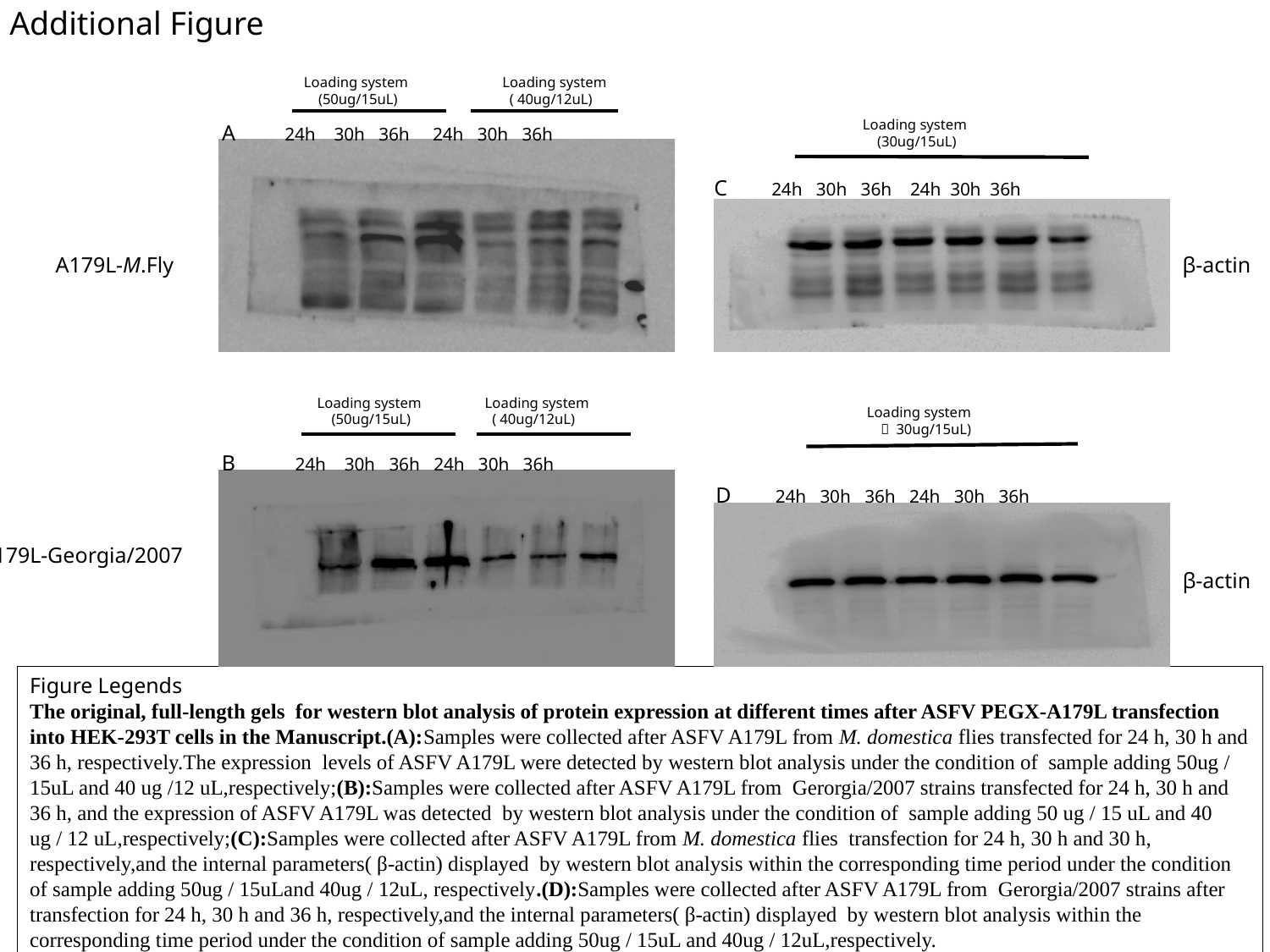

Additional Figure
 Loading system
 (50ug/15uL)
Loading system
 ( 40ug/12uL)
A 24h 30h 36h 24h 30h 36h
 Loading system
 (30ug/15uL)
 C 24h 30h 36h 24h 30h 36h
A179L-M.Fly
β-actin
 Loading system
 (50ug/15uL)
Loading system
 ( 40ug/12uL)
B 24h 30h 36h 24h 30h 36h
 Loading system
 （ 30ug/15uL)
D 24h 30h 36h 24h 30h 36h
β-actin
A179L-Georgia/2007
Figure Legends
The original, full-length gels for western blot analysis of protein expression at different times after ASFV PEGX-A179L transfection into HEK-293T cells in the Manuscript.(A):Samples were collected after ASFV A179L from M. domestica flies transfected for 24 h, 30 h and 36 h, respectively.The expression levels of ASFV A179L were detected by western blot analysis under the condition of sample adding 50ug / 15uL and 40 ug /12 uL,respectively;(B):Samples were collected after ASFV A179L from Gerorgia/2007 strains transfected for 24 h, 30 h and 36 h, and the expression of ASFV A179L was detected by western blot analysis under the condition of sample adding 50 ug / 15 uL and 40 ug / 12 uL,respectively;(C):Samples were collected after ASFV A179L from M. domestica flies transfection for 24 h, 30 h and 30 h, respectively,and the internal parameters( β-actin) displayed by western blot analysis within the corresponding time period under the condition of sample adding 50ug / 15uLand 40ug / 12uL, respectively.(D):Samples were collected after ASFV A179L from Gerorgia/2007 strains after transfection for 24 h, 30 h and 36 h, respectively,and the internal parameters( β-actin) displayed by western blot analysis within the corresponding time period under the condition of sample adding 50ug / 15uL and 40ug / 12uL,respectively.
